# The Influence of Temperature and Stoichiometry on the Optical Properties of CdSe Nanoplatelets

**DOI:** 10.3390/nano14221794

**Published:** 2024-11-08

**Authors:** Yerkebulan Koshkinbayev, Aigerim Ospanova, Aizhan Akhmetova, Turlybek Nurakhmetov, Asset Kainarbay, Keleshek Zhangylyssov, Sergey Dorofeev, Alexander Vinokurov, Sergei Bubenov, Dulat Daurenbekov

**Affiliations:** 1 Institute of Physical and Technical Sciences, L.N. Gumilyov Eurasian National University, 13 Kazhymukan st., 010000 Astana, Kazakhstan; koshkinbayev17@gmail.com (Y.K.);; 2 Department of Chemistry, Lomonosov Moscow State University, 1-3 Leninskie Gory, Moscow 119991, Russia

**Keywords:** semiconductor, 2D structure, CdSe, nanoplatelets, colloidal synthesis, total reflection X-ray fluorescence

## Abstract

Colloidal quasi-two-dimensional cadmium chalcogenide nanoplatelets have attracted considerable interest due to their narrow excitonic emission and absorption bands, making them promising candidates for advanced optical applications. In this study, the synthesis of quasi-two-dimensional CdSe NPLs with a thickness of 3.5 monolayers was investigated to understand the effects of synthesis temperature on their stoichiometry, morphology, and optical properties. The NPLs were synthesized using a colloidal method with temperatures ranging from 170 °C to 210 °C and optimized precursor ratios. Total reflection X-ray fluorescence (TXRF) analysis was employed to determine stoichiometry, while high-resolution transmission electron microscopy (HRTEM) and UV-Vis spectroscopy and photoluminescence spectroscopy were used to analyze the structural and optical characteristics. The results showed a strong correlation between increasing synthesis temperature and the enlargement of nanoscroll diameters, indicating dynamic growth. The best results in terms of uniformity, stoichiometry, and optical properties were achieved at a growth temperature of 200 °C. At this temperature, no additional optical bands associated with secondary populations or hetero-confinement were observed, indicating the high purity of the sample. Samples synthesized at lower temperatures exhibited deviations in stoichiometry and optical performance, suggesting the presence of residual organic compounds.

## 1. Introduction

Colloidal semiconductor nanomaterials have garnered significant attention due to their unique optical and electronic properties [1,2]. Among these, quasi-two-dimensional nanoplatelets (NPLs) made of cadmium selenide (CdSe) stand out because of their atomically controlled thickness and large lateral dimensions, which differentiate them from zero-dimensional quantum dots and one-dimensional nanorods. The quasi-two-dimensional structure of NPLs gives them enhanced absorption and emission characteristics, including extremely narrow photoluminescence (PL) lines and high oscillator strengths, making them highly promising for various optoelectronic applications, such as photodetectors [3,4,5], light-emitting diodes [6,7,8,9], and lasers [10,11].

Advancements in the synthesis of colloidal NPLs have enabled precise control over their thickness at the monolayer level [2,12,13], and aspect ratios [14], which allows for fine-tuning of their optoelectronic properties. However, challenges remain in fully understanding the complex mechanisms behind NPL formation. Since the first synthesis of NPLs was reported in 2008 [1], significant efforts have been made to elucidate the growth mechanisms of these structures [2,15,16,17].

Like zero-dimensional quantum dots and quasi-one-dimensional nanorods, colloidal quasi-two-dimensional NPLs are metastable crystal states, and their synthesis involves complex multicomponent reaction mixtures. Standard synthesis methods for NPLs often use cadmium acetate dihydrate, the degree of hydration of which affects the properties of the final product [14]. Additionally, components such as trioctylphosphine [10,18] (90%) for preparing the anionic precursor and oleic acid [19] (90%) are used, both of which may contain unknown impurities that could introduce catalytic effects during the synthesis. In some techniques 1-octadecene (90%) is employed to prepare selenium solutions, where the reaction pathways can be difficult to control [19,20,21]. Therefore, as an alternative to deep studies of these complex multicomponent systems, standardized synthesis protocols can be established.

It is well established that synthesis conditions, including temperature, reaction time, precursor concentration, and other parameters, have a direct impact on the structure, morphology, and stoichiometry of NPLs. In a study by Singh and co-workers [22], the researchers determined the stoichiometry of 5 ML CdSe NPLs with a wurtzite structure using Rutherford backscattering spectroscopy (RBS). They calculated the Cd-to-Se ratio to be 1.21 ± 0.02, which was slightly lower than the theoretical value of 1.255, as estimated by the authors. This value was based on a model that assumed the presence of an additional 0.5 monolayer of cadmium to compensate for surface coordination defects. In the study by Sun and Buhro [23] the stoichiometry of 3.5 ML CdSe NPLs with a zinc blende crystal structure was determined using energy-dispersive X-ray spectroscopy (EDS), resulting in a ratio of 1.26 ± 0.03, slightly lower than the theoretical value of 1.33 due to the presence of an additional half monolayer of cadmium on the surface. It is likely that a cadmium deficiency in the surface layers of NPLs affects surface stress distribution, morphology, and optical properties.

In this work, we present a method for synthesizing 3.5 ML CdSe NPLs and provide a comprehensive characterization of the obtained samples in terms of Cd and Se yield, stoichiometry, morphology, and optical properties. The absolute masses of Cd and Se were determined using total reflection X-ray fluorescence (TXRF) analysis. The Cd and Se yields were calculated to analyze the stoichiometry and its correlation with the structure and optical properties of NPLs. The structure of NPLs was examined using high-resolution transmission electron microscopy (HRTEM), while their optical properties were investigated through UV-Vis spectroscopy and photoluminescence spectroscopy.

## 2. Materials and Methods

Chemicals: Cadmium acetate dihydrate (Cd (CH_3_COO)_2_·2H_2_O), oleic acid (OA, Sigma-Aldrich, 90%, St. Louis, MI, USA), selenium powder (Se, Sigma-Aldrich, 99.99%, Tokyo, Japan), trioctylphosphine (TOP, Sigma-Aldrich, 90%, Tokyo, Japan), 1-octadecene (ODE, Sigma-Aldrich, 90%, Doha, Qatar), hexane, acetone.

1M TOPSe. To prepare a 1 M solution of TOPSe, 0.16 g of selenium powder was mixed with 2 mL of TOP until fully dissolved, then the solution was stored for future use.

Synthesis of CdSe NPLs: The synthesis was adapted from [24,25] with minor modifications. In a 50 mL two-neck flask, 0.5 mmol (0.13 g) of Cd (Ac)_2_·2H_2_O was mixed with 0.25 mmol (80 μL) of OA and 10 mL of ODE. The mixture was degassed at 60 °C for 1 h. The temperature was then increased to 160 °C. At 120 °C, a solution of 125 μL of 1M TOPSe, diluted with 250 μL of ODE, was injected. The reaction mixture was left to react at 160 °C for 1 h, then cooled to room temperature. At 150 °C, 1 mL of OA was added. The resulting NPLs were washed with acetone and centrifuged at 5000 rpm for 5 min. This purification process was repeated twice. Finally, the NPLs were redispersed in hexane.

Characterization Techniques: Absorption spectra were measured in hexane dispersions using a V-770 spectrophotometer (Jasco, Tokyo, Japan) over a wavelength range of 200–800 nm. A CM2203 spectrofluorimeter (Solar, Minsk, Belarus) was used to study photoluminescence (PL) spectra and PL excitation spectra.

The composition of the samples was determined using a TXRF spectrometer (S2 Picofox, Bruker, Berlin, Germany) (excitation by Mo Kα radiation, voltage 50 kV). The NPL dispersions were dried on sapphire substrates, and the absolute masses of elements were determined.

HRTEM studies were carried out using a JEM-2100 (JEOL, Tokyo, Japan) electron microscope equipped with a LaB_6_ electron gun and an 11-megapixel CCD camera (Quemesa, Olympus, Tokyo, Japan). The accelerating voltage was set to 200 kV. The hexane dispersions of NPLs were deposited on carbon-coated copper grids and dried. The grids were plasma-cleaned in an argon atmosphere to remove organic material before analysis. The lateral dimensions and scroll radii of the NPLs were manually measured on the micrographs using Image-Pro Plus 6.0 software. Fast Fourier transform (FFT) patterns of selected areas in the micrographs were obtained using the ImageJ program.

Quantitative IR spectroscopy was performed as follows. A hexane sol of NPLs was intensely sonicated over 5 min. Then, amid sonication 200 µL of sol were removed with a micropipette and added to pre-ground KBr powder in a plastic beaker. The beaker was sonicated for 5 s, then centrifuged for 1 min at 2.5 × 10^4^× *g*, and the organic supernatant was discarded. The resultant yellow powder was then ground with additional KBr and pressed into a pellet. This sequence was carried out to combat non-uniformity in the distribution of the substance in the probe, which is known to be the primary source of error in quantitative IR. IR spectra and UV-Vis spectra were averaged over 4 positions (produced by 90° rotations of the pellet in the sample holder) for each sample. Scattering was significant, especially in the UV-Vis region; however, the uniquely sharp absorption features of NPLs were able to mitigate this adverse effect in the manner, as shown in the Supplementary Information. With the ratio of the intensity (absorbance units) of carboxylate band in the IR region (wavenumber 1550 cm^−1^) to the intensity of heavy hole (HH) absorption feature (wavelength ~460 nm), the samples could be compared in terms of their carboxylate content normalized to the Cd_4_Se_3_ formula unit of the inorganic cores. A relative error as low as 4% was achieved for the technique.

## 3. Results and Discussion

*Growth of Colloidal CdSe NPLs.* For the synthesis of a CdSe NPL ensemble with a thickness of 3.5 monolayers (ML), we used a colloidal growth protocol employing a mixture of octadecene, oleic acid, and cadmium acetate dihydrate. Trioctylphosphine selenide was used as the selenium source, and cadmium acetate dihydrate served as the cadmium precursor.

**Figure 1:**
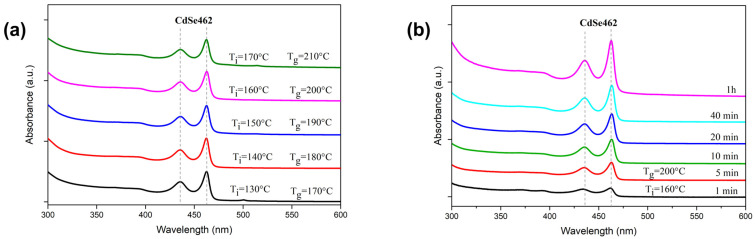
(**a**) The effect of temperature on the absorption spectra of CdSe NPLs, obtained after 1 h of growth at various injection temperatures (T_i_) and growth temperatures (T_g_). (**b**) Evolution of absorption spectra recorded at different times for CdSe NPLs synthesized with growth parameters T_i_ = 160 °C and T_g_ = 200 °C.

We analyzed the effect of temperature on the growth of CdSe NPLs. The absorption spectra obtained at different temperatures are shown in Figure 1a, where T_i_ is the injection temperature, and T_g_ is the growth temperature. The temperature difference between injection and growth in this series of experiments was 40 degrees. Across the entire range of growth temperatures (170–210 °C), a distinct ensemble of CdSe462 NPLs was observed, where the first excitonic transition occurs at 462 nm. This corresponds to 3.5 ML CdSe NPLs [26]. Starting from the injection temperature, we then increased the temperature to T_g_ over the course of 5–10 min and maintained it constant until the end of the growth period (1 h). During the growth, only absorption bands of the CdSe462 NPL ensemble were detected, as shown in Figure 1b, where the NPLs were grown at T_g_ = 200 °C.

Then, we analyzed the effect of growth temperature on the yield of Cd and Se. The stoichiometry of the CdSe NPLs was determined using TXRF analysis (Table S1). Using the absolute mass values of Cd and Se obtained from elemental analysis, we calculated the concentrations and yields of Cd and Se in the samples. The Cd:Se atomic ratio in the NPLs was calculated from the Cd and Se yields in the samples. As shown in the table, the stoichiometry in the CdSe180 and CdSe190 samples is imbalanced, while in the samples synthesized at higher temperatures (CdSe200 and CdSe210), the Cd:Se ratio is closer to the expected value of 1.33 for CdSe NPLs with a thickness of 3.5 ML, which is consistent with the models described by Sun and Buhro [23]. According to their results, CdSe NPLs with a similar thickness should exhibit a stoichiometry of 1.33, due to an additional half-monolayer of cadmium atoms on the surfaces, leading to an increased cadmium content relative to selenium. In the CdSe180 and CdSe190 samples, an excess of Cd was observed. We hypothesize that this is due to the presence of free Cd oleate in the solutions or absorbed on the surface of NPLs.

This is corroborated by quantitative IR results. The lowest carboxylate content was found for the CdSe170 sample, which is incidentally Cd-deficient. CdSe200 has 1.5 times more oleate, and when adjusted for NPL concentration, CdSe180 and CdSe190 have 3.1 and 5.6 times more oleate than sample CdSe170, respectively.

**Figure 2:**
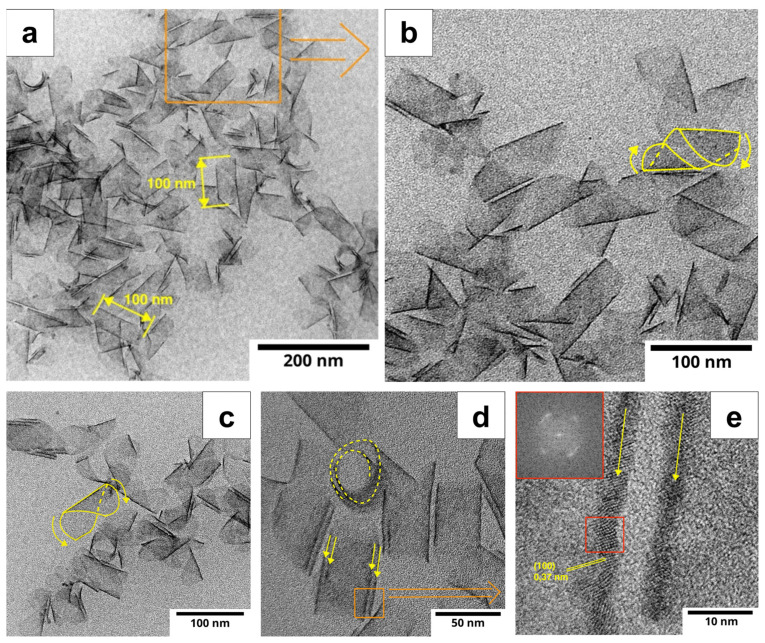
Overview HRTEM images at various magnifications of the synthesized scroll-like CdSe NPLs grown at 200 °C (**a**–**c**) and 180 °C (**d**,**e**). The inset shows the FFT pattern of the area marked by the red square. The double yellow arrows in (**d**,**e**) indicate the double walls of the rolled-up NPLs. Single arrows in (**b**,**c**) indicate the direction of nanoscroll folding.

*Morphology.* The morphology of the NPLs was investigated using HRTEM analysis. The HRTEM images of the synthesized 3.5 ML CdSe NPLs (Figure 2a−d) show an ensemble of scroll-like nanoplatelets. The scroll-like nature of the NPLs is preserved across all samples synthesized at different temperatures. The average lateral size along the twisting axis is about 100 nm. The NPLs are typically twisted 1–2 times, which can be seen from the edge of the nanosheet and as two edge lines on each side of the NPL, marked with yellow arrows in the TEM images (Figure 2d). Some NPLs were not fully twisted, as shown in Figure 2c.

The crystalline nature of the NPLs was confirmed by HRTEM data in the form of lattice fringes in the image of the NPL side edge (Figure 2e), where the interplanar spacing is approximately 3.7 Å. This value is comparable to the X-ray diffraction data for the wurtzite phase of CdSe, where the interplanar spacing for the (100) plane is 3.72 Å. Thus, it can be concluded that the (100) plane orientation dominates in the CdSe NPLs studied, which is consistent with known data on the structure of this material. 

**Figure 3:**
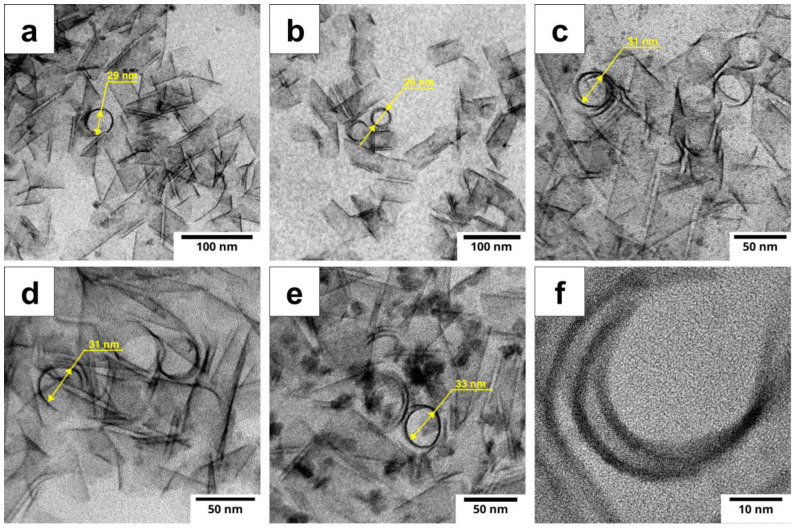
Edge-on images of nanoscrolls synthesized at temperatures (**a**) 170 °C, (**b**) 180 °C, (**c**) 190 °C, (**d**) 200 °C, (**e**) 210 °C, obtained by HRTEM. (**f**) HRTEM cross-section image of a single scroll-like CdSe NPLs.

Since the nanoplatelets have a scroll-like structure, accurately determining their lateral dimensions is a challenging task and may be practically impossible. Therefore, analyzing the scroll diameter becomes the only method to assess the growth of nanoplatelets and understand the dynamics of their formation. The TEM image analysis, in which the nanoscrolls were viewed from the edge, showed that with increasing NPL growth temperature, a dynamic increase in the outer scroll diameter is observed (Figure 3). For samples synthesized at 170 °C and 180 °C, the scroll diameters were 29 nm. In the samples synthesized at 190 °C and 200 °C, the average diameter increased to 32 nm. The maximum diameter was observed in the sample synthesized at 210 °C, reaching 33 nm. The deviation from the average diameter for all samples did not exceed 1 nm, indicating a high uniformity of the synthesized nanoscrolls. These data indicate that the morphology of the nanoscrolls depends on the synthesis temperature—the surface stresses that determine the scroll curvature radius change systematically with increasing temperature. This is important for controlling their dimensions during the synthesis of nanomaterials and may significantly influence their subsequent use in optoelectronic applications. 

**Figure 4:**
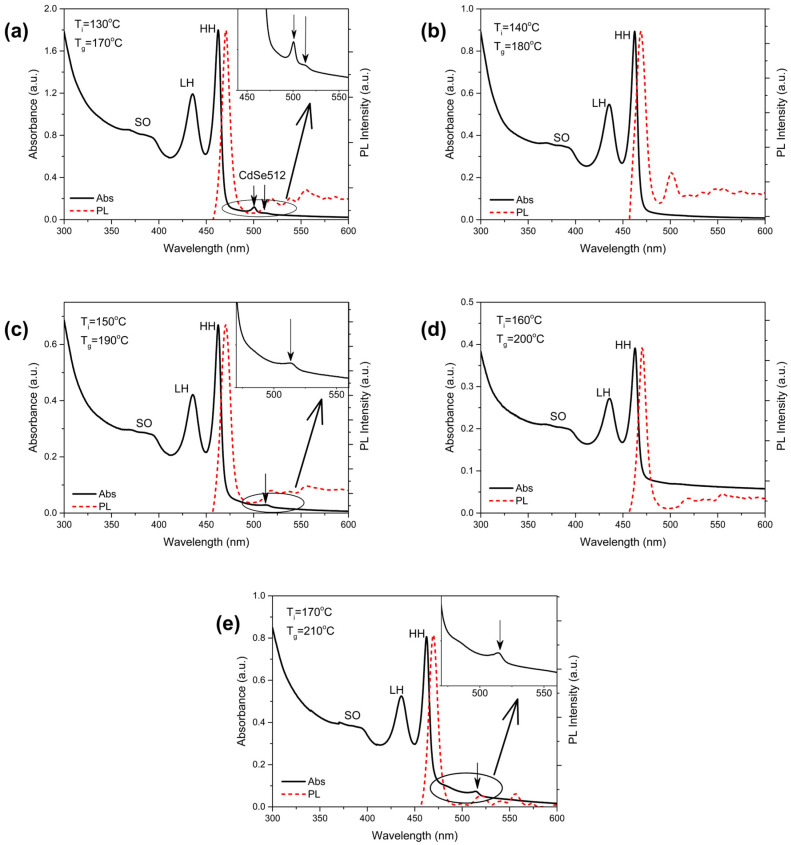
Absorption and photoluminescence spectra of CdSe NPLs synthesized at different temperatures: (**a**) 170 °C; (**b**) 180 °C; (**c**) 190 °C; (**d**) 200 °C; (**e**) 210 °C. The absorption spectra are shown as solid black lines, while the photoluminescence is depicted as dashed red lines.

*Optical Properties.* The optical properties of the NPLs were studied in the form of hexane sols using absorption, photoluminescence (PL), and PL excitation spectroscopy. As shown in Figure 4a–e, the absorption spectra of all samples exhibit narrow excitonic transitions at 462, 435, and 393 nm, corresponding to HH, light holes (LH), and spin–orbit holes (SO), respectively, which are in good agreement with the literature data [18,27]. The formation of nanoscrolls preserves the excitonic structure in the absorption spectra; however, significant scattering is observed, which is attributed to the large size of the NPLs. Even at a low concentration of NPLs (about 0.01 in absorbance), the scattering effect persists. Against the background of scattering, the absorption spectra of samples synthesized at 170 °C, 190 °C, and 210 °C also exhibit weak absorption bands. In the CdSe170 NPL sample, these peaks appear at 512 and 500 nm, while for samples synthesized at 190 °C and 210 °C, they are observed at 512 and 514 nm, respectively. This indicates the presence of an ensemble of NPLs with a thickness of 4.5 ML alongside the main population of 3.5 ML NPLs. Similar effects have been observed in the work of other authors [16,17].

When studied in the form of pellets pressed with KBr, the spectral positions of HH and LH absorption maxima showed gradual blue-shift with higher synthetic temperatures (Table 1). We suggest that, upon drying, the same monotonic variation in mechanical stresses affects both the nanoscroll diameters and absorption maxima positions. The variation in stress is likely the result of different concentration of surface defects (stoichiometry).

A distinct excitonic photoluminescence (PL) band at 470 nm was observed in the PL spectra, with a Stokes shift of 8 nm. Defect-related emission bands were detected at 520, 540, and 555 nm for samples synthesized at 170 °C, 190 °C, 200 °C, and 210 °C, while in the CdSe180 sample, these bands appeared at 533 and 555 nm. Additionally, a band with a peak at 503 nm was observed in the CdSe180 sample. This peak was not accompanied by corresponding absorption bands, suggesting that it may be related to hetero-confinement. Similar deviations have been reported in a study on CdTe NPLs [28], where such effects were attributed to the presence of layers with different thicknesses on the same nanoplatelet. In contrast, for the samples synthesized at 170 °C, 190 °C, and 210 °C, it can be concluded that this phenomenon is not related to hetero-confinement, as the intensity of the peaks corresponding to the 3.5 ML NPLs significantly exceeds that of the 4.5 ML peaks. This indicates the presence of two independent NPL ensembles rather than a combination of different thicknesses within the same nanoplatelet.

The analysis of the optical properties of the NPLs, combined with their stoichiometric assessment, indicates that the NPLs with the most optimal characteristics—characterized by minimal ensemble variation and precise stoichiometry—are achieved at a growth temperature of 200 °C. This sample exhibits the highest uniformity in NPL thickness and shows no additional bands associated with secondary populations or hetero-confinement.

The full width at half maximum (FWHM) did not change with variations in the synthesis temperature. For excitonic absorption related to heavy holes, the FWHM was 9 nm, while for light holes, it was 12 nm. Additionally, for excitonic luminescence, the FWHM was 10 nm.

**Figure 5:**
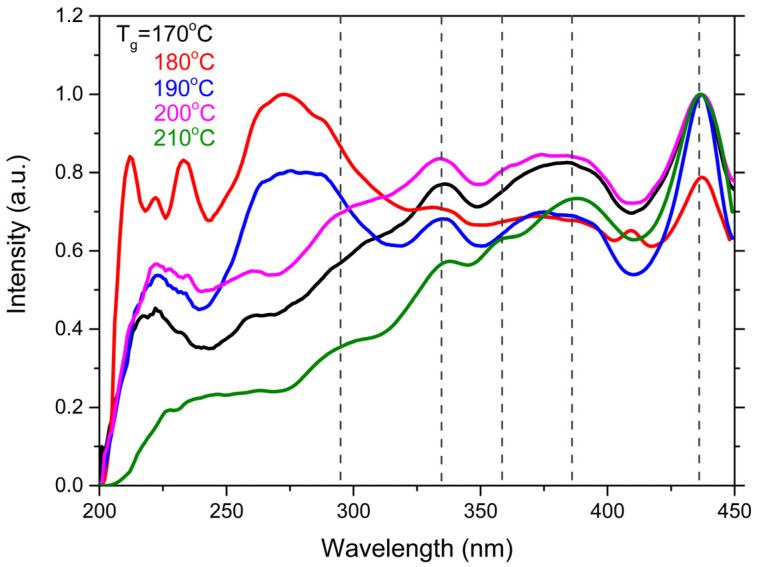
Photoluminescence excitation spectra of CdSe NPLs synthesized at different temperatures.

As the excitonic photoluminescence remained consistent across all samples, we proceeded to analyze the photoluminescence excitation (PLE) spectra. The excitation spectra were measured for the luminescence band at 470 nm. As shown in Figure 5, the variation in the PLE spectra suggests a reorganization of electronic transitions without affecting the emissive properties.

In the samples with disrupted stoichiometry (CdSe180, CdSe190), the photoluminescence excitation spectra exhibit distortions in the 200–300 nm range. These peaks may be attributed to residual organic material that did not fully react and remained on the surface of the nanoplatelets after synthesis and purification. Organic compounds, such as residual ligands (oleic acid, trioctylphosphine, etc.), typically absorb light in the UV range, and their presence on the surface can introduce additional peaks in the excitation spectra. However, the precise correlation between the excitation spectra and the electronic structure of the NPLs remains an open question.

Table 1 presents the results of the analysis of the synthesized CdSe nanoplatelets, covering parameters such as stoichiometry, crystalline phase, average scroll diameter, and their optical properties.

The stoichiometric analysis reveals the cadmium-to-selenium ratio in each sample. In the samples synthesized at 200 °C and 210 °C, this ratio approaches the theoretically expected value of 1.33 for nanoplatelets with a thickness of 3.5 ML, demonstrating effective control over the synthesis process. In contrast, at lower temperatures (180 °C and 190 °C), a significant deviation from the theoretical stoichiometry is observed, with an excess of cadmium.

All samples exhibit a wurtzite crystalline phase, indicating the stability of this structure for CdSe NPLs across the growth temperature range of 170 °C to 210 °C. However, despite the consistent crystalline phase, the scroll diameters increase noticeably with rising temperature: from 29 nm at 170 °C and 180 °C to 33 nm at 210 °C.

The optical characteristics of the samples, including the wavelengths of heavy- and light-hole excitonic transitions and photoluminescence at 470 nm, remain stable across all temperatures. This suggests that the primary excitonic transitions are preserved in the spectra of all samples, regardless of stoichiometric variations.

A key aspect is the state of the electronic structure, as assessed through photoluminescence excitation (PLE) spectra. In the samples synthesized at 170 °C, 200 °C, and 210 °C, the excitation spectra are undistorted, indicating an ordered electronic structure. In contrast, significant distortions are observed in the PLE spectra of samples synthesized at 180 °C and 190 °C.

An analysis of the optical properties indicates that NPLs with the highest uniformity in thickness are achieved at a growth temperature of 200 °C. This sample shows no additional bands associated with secondary ensembles or hetero-confinement.

## 4. Conclusions

In this study, a method for synthesizing quasi-two-dimensional CdSe nanoplatelets (NPLs) with a thickness of 3.5 monolayers was applied, and their stoichiometry was analyzed in relation to their structural and optical properties. Using a colloidal synthesis method with controlled growth temperatures ranging from 170 °C to 210 °C and optimized precursor ratios, scroll-like nanoplatelets with a high uniformity and a single wurtzite crystalline structure were obtained. The experimental results demonstrated that the synthesis temperature significantly affects the morphology and stoichiometry of the nanoplatelets. A direct correlation was observed between increasing growth temperature and the enlargement of the nanoscroll diameters, reflecting the dynamic growth of the NPLs. The best results in terms of stoichiometry, morphology, and optical properties were obtained at a growth temperature of 200 °C. This sample shows no additional bands associated with secondary ensembles or hetero-confinement.

## Figures and Tables

**Table 1:** Summary of data for the analysis of the CdSe NPL samples.

Method		TXRF	TEM		UV-Vis Spectroscopy in KBr Pellet		Luminescence Spectroscopy	
	Characteristics	Stoichiometry	Crystalline Phase	Average Diameter of Nanoscrolls (nm)	HH	LH	PL	PLE
Sample								
CdSe170		1.14	wurtzite	29	461.7 nm	435.6 nm	470 nm	✓
CdSe180		2.70	wurtzite	29	460.6 nm	434.5 nm	470 nm	✕
CdSe190		2.42	wurtzite	31	460.1 nm	434.5 nm	470 nm	✕
CdSe200		1.31	wurtzite	31	459.6 nm	434.1 nm	470 nm	✓
CdSe210		1.29	wurtzite	33	459.4 nm	434.1 nm	470 nm	✓

## Data Availability

The data underlying the results presented in this paper are not publicly available at this time but may be obtained from the authors upon reasonable request.

## Supplementary Materials

The following supporting information can be downloaded at: https://www.mdpi.com/article/10.3390/nano14221794/s1, Table S1: The results of elemental analysis (TXRF) are presented as the absolute masses of elements in the deposited sample, the yield of cadmium and selenium, and the stoichiometry. Figure S1. Visible absorption spectra of the samples CdSe170 and CdSe200 studied in the form of pellets in KBr. Figure S2. Graphical method employed to correct scattering effect on the intensity and position of HH and LH maxima of NPLs in the form of pellets in KBr.
