# Supplementary material for: The Influence of Temperature and Stoichiometry on the Optical Properties of CdSe Nanoplatelets
Source: Nanomaterials (Basel). 2024 Nov 8;14(22):1794. doi: 10.3390/nano14221794 (PMC11597073; doi:10.3390/nano14221794)
Supplement: Supplementary file 1 [file nanomaterials-14-01794-s001.zip › nanomaterials-3273727-supplementary.pdf]

Supplementary Information fo

The Influence of Temperature and Stoichiometry on the Optical Properties of CdSe Nanoplatelets

Yerkebulan Koshkinbayev [1], Aigerim Ospanova [1], Aizhan Akhmetova [1], Turlybek Nurakhmetov [1], Asset Kainarbay [1], Keleshek Zhangylyssov [1], Sergey Dorofeev [2], Alexander Vinokurov [2], Sergei Bubenov [2,*] and Dulat Daurenbekov [1,*]

[1] Institute of Physical and Technical Sciences, L.N. Gumilyov Eurasian National University, 13 Kazhymukan st., 010000 Astana, Kazakhstan; koshkinbayev17@gmail.com (Y.K.);

[2] Department of Chemistry, Lomonosov Moscow State University, 1-3 Leninskie Gory, Moscow 119991, Russia;

* Correspondence: bubenovss@my.msu.ru (S.B.); 900902399020@enu.kz (D.D.)

Table S1. The results of elemental analysis (TXRF) are presented as the absolute masses of elements in the deposited sample, the yield of cadmium and selenium, and the stoichiometry.

Sample	m_{Cd} [ng]	m_{Se} [ng]	n_{Cd} [µmol]	n_{Se} [µmol]	Stoichiometry n_{Cd}/n_{Se}
CdSe170	153.65	95.013	13.00	11.44	1.14
CdSe180	745.0	193.49	15.77	5.83	2.70
CdSe190	407.3	118.193	22.42	9.26	2.42
CdSe200	163.1	87.132	33.66	25.60	1.31
CdSe210	176.34	96.23	24.60	19.11	1.29

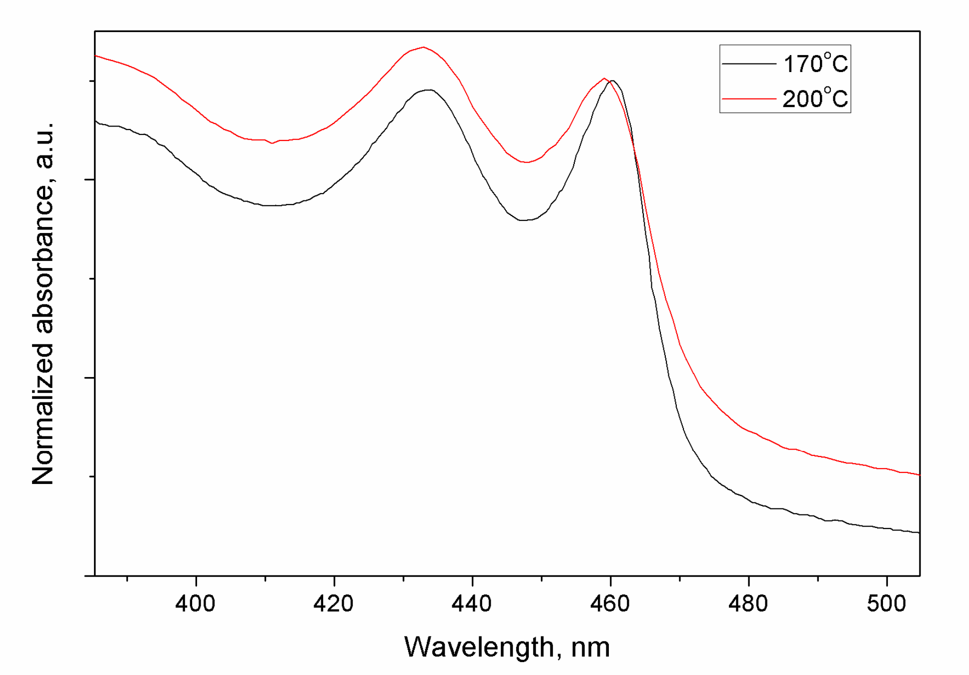

Figure S1. Visible absorption spectra of the samples CdSe170 and CdSe200 studied in the form of pellets in KBr

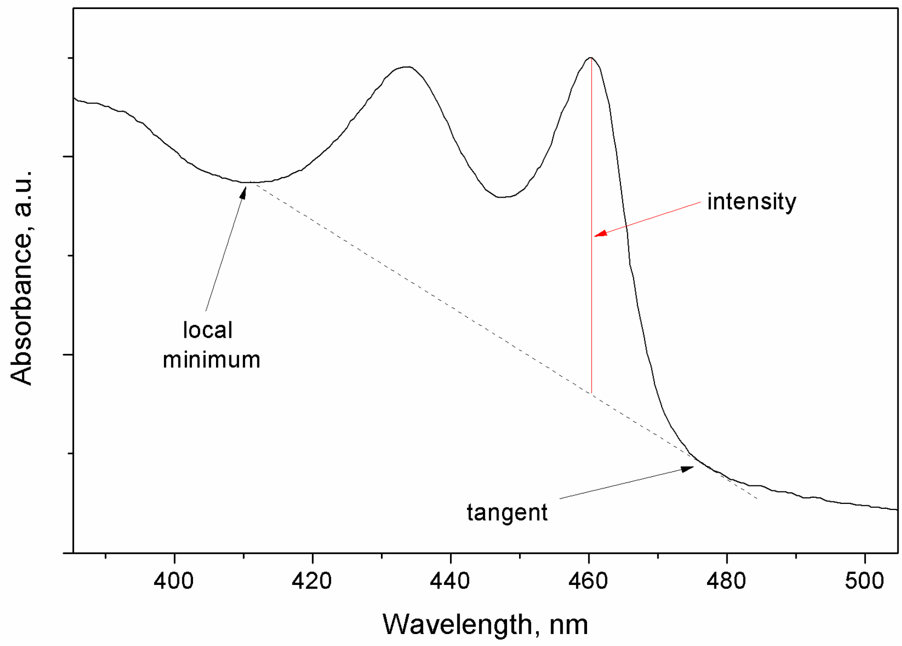

Figure S2. Graphical method employed to correct scattering effect on the intensity and position of HH and LH maxima of NPLs in the form of pellets in KBr
